# Stability of Oral Liquid Dosage Forms in Pediatric Cardiology: A Prerequisite for Patient’s Safety—A Narrative Review

**DOI:** 10.3390/pharmaceutics15041306

**Published:** 2023-04-21

**Authors:** Carmen-Maria Jîtcă, George Jîtcă, Bianca-Eugenia Ősz, Amalia Pușcaș, Silvia Imre

**Affiliations:** 1 Doctoral School of Medicine and Pharmacy, I.O.S.U.D., George Emil Palade University of Medicine, Pharmacy, Science, and Technology of Târgu Mureș, 540139 Târgu Mureș, Romania; carmenrusz20@gmail.com; 2 Department of Pharmacology and Clinical Pharmacy, Faculty of Pharmacy, George Emil Palade University of Medicine, Pharmacy, Science, and Technology of Târgu Mureș, 540139 Târgu Mureș, Romania; bianca.osz@umfst.ro; 3 Department of Biochemistry, Faculty of Pharmacy, George Emil Palade University of Medicine, Pharmacy, Science, and Technology of Târgu Mureș, 540139 Târgu Mureș, Romania; amalia.puscas@umfst.ro; 4 Department of Analytical Chemistry and Drug Analysis, Faculty of Pharmacy, George Emil Palade University of Medicine, Pharmacy, Science, and Technology of Târgu Mureș, 540139 Târgu Mureș, Romania; silvia.imre@umfst.ro

**Keywords:** stability, oral liquid, dosage form, pediatric, cardiology

## Abstract

The development of safe and effective pediatric formulations is essential, especially in therapeutic areas such as pediatric cardiology, where the treatment requires multiple dosing or outpatient care. Although liquid oral dosage forms are considered the formulation of choice given the dose flexibility and acceptability, the compounding practices are not endorsed by the health authorities, and achieving stability can be problematic. The purpose of this study is to provide a comprehensive overview of the stability of liquid oral dosage forms used in pediatric cardiology. An extensive review of the literature has been performed, with a particular focus on cardiovascular pharmacotherapy, by consulting the current studies indexed in PubMed, ScienceDirect, PLoS One, and Google Scholar databases. Regulations and guidelines have been considered against the studies found in the literature. Overall, the stability study is well-designed, and the critical quality attributes (CQAs) have been selected for testing. Several approaches have been identified as innovative in order to optimize stability, but opportunities to improve have been also identified, such as in-use studies and achieving dose standardization. Consequently, the information gathering and the results of the studies can be translated into clinical practice in order to achieve the desired stability of liquid oral dosage forms.

## 1. Introduction

The lack of adequate pharmaceutical formulations for children leading to off-label use of drugs is a topic well-documented in the literature among healthcare specialists, regulators, and the academic community [1,2,3,4]. The importance of this topic can hardly be overestimated, but despite the efforts, there are some therapeutic areas, such as cardiovascular diseases in children, that are completely overlooked. Worldwide, initiatives are being taken in order to develop pediatric medicines, and the main regulators, namely, European Medicines Agency (EMA) and Food and Drug Administration (FDA) [5], have defined requirements and issued guidelines for the industry [6]. In the European Union (EU), the necessity of developing pediatric drugs has been regulated by the EMA through the *Paediatric Regulation* in 2007, whereas in the United States (US), pediatric drug development followed the *Pediatric Research Equity Act* (PREA) founded in 2003 and the *Best Pharmaceuticals for Children Act* (BPCA) founded in 2002, both regulated by the FDA.

A 10-year follow-up report of the *Paediatric Regulation* indicates a clear upwards trend, with 267 new medicines and 43 new pharmaceutical forms for children’s use [7]. The strong interest in this therapeutic area is also reflected in the number of Paediatric Investigation Plans (PIPs) submitted by pharmaceutical companies up until 2017 [8,9]. The World Health Organization (WHO) issues Model Lists of Essential Medicines for Children every two years in order to direct the research regarding certain drug substances that are considered essential for pediatric treatment. The eighth list from 2021 comprises enalapril, digoxin, furosemide, and dopamine [10]. The EMA also issued an inventory of pediatric therapeutic needs [11]. In the cardiovascular therapeutic area, several substances, such as clonidine, atenolol, bisoprolol, carvedilol, sotalol, amiodarone, flecainide, and nicardipine have been listed, with a particular focus on information required regarding safety and pharmacokinetics and the need for developing age-appropriate formulations [11,12,13]. Nonetheless, there are still few medications that have been approved for children’s use, especially regarding age-appropriate formulations. This aspect has also been emphasized by del Moral-Sanchez et al., whose findings indicate that for the cardiovascular therapeutic area, only 4.2% of oral dosage forms are available. Concerning all of the substances analyzed, it has been emphasized that despite overall high availability, few drug dosage formulations are age-appropriate [14]. It should, however, be noted that inconsistencies have also been identified, in the sense that some of the developed age-appropriate formulations are only developed to meet special pediatric requirements, e.g., an oral solution of propranolol for infantile hemangioma.

Based on the above considerations, it can be easily assumed that in the pediatric cardiology therapeutic area, the treatment is mostly constituted by off-label or even unlicensed practices, represented by the manipulation of the adult-approved dosage form. Current practices involve crushing the commercially available tablets or using the content of capsules and mixing them with a diluent in order to obtain a powder of a suitable dosage, which is then added to baby food or proper liquid for administration [15,16,17]. These practices are susceptible to dosing errors and can even disregard the existence of potentially harmful excipients that are inadequate for children’s use [16,18]. Another practice that involves diluting the commercially available injectable form for oral administration has also been mentioned in the literature [15,17,19].

There is an increasing interest in developing safe and effective pediatric formulations. Both solid and liquid oral dosage forms have been extensively studied in the literature [20,21,22,23,24], indicating suitable use in children. Several solid oral dosage forms have been studied to date, such as orodispersible tablets, mini-tablets, and chewable tablets [8,25,26,27]. While solid formulations demonstrated improved stability which supported longer shelf-lives, pediatric oral liquid dosage forms are dose-flexible and easy to swallow by the patients [20,28]. However, these solid oral dosage forms are not approved, and several extensive studies are required in order to establish their safe use in all age groups. According to EMA’s reflection paper on formulations of choice for the pediatric population, oral liquid dosage forms represent the form of choice for infants and toddlers (1 month–2 years) and young children (2–5 years) [29,30], especially in children less than 12 years old [18], and this is supported by current literature findings. The most common forms used are solutions, suspensions, and syrups. Solutions are preferred over suspensions due to better oral acceptance and the tendency of the latter to sediment, and also because insufficient redispersion makes them susceptible to dosing errors [30,31].

Some of the limitations of liquid dosage forms include the stability and the difficulty of obtaining controlled release formulations, which require multiple-day dosing, exposure to undesirable excipients, extra palatability, and higher costs [20,32]. The need for improving the liquid formulations and also the standardization of dosing devices in order to maintain dose flexibility and children’s acceptability have been emphasized [20,33,34]. Considering these aspects, pharmaceutical compounding of oral liquid dosage forms is challenging and can vary a lot in terms of provenance of Active Pharmaceutical Ingredient (API), excipients, preparation methods, and the dosage strength obtained. It is fairly stipulated in the literature that these compounded formulations are exempted from Good Manufacturing Practice (GMP), and the testing to assess product quality is inconsistent [35]. Therefore, rigorous stability studies should be conducted for every particular formulation, on a case-by-case basis.

In terms of opportunity, some Marketing Authorization Holders (MAHs) have already initiated actions towards pediatric drug administration by employing the so-called “industry-verified preparations”. This term refers to several well-defined and verified steps contained in the package leaflet in order to prepare an oral liquid from marketed tablets or capsules that can ensure the stability and dose uniformity of the preparation [9].

Many studies related to the stability of oral liquid dosage forms in the cardiovascular therapeutic area are present in the literature, which emphasize a continuous need for this formulation in order to cover the lack of approved medication. These findings are consistent with those of Belayneh and Tessema 2021, who conducted a systematic review of the stability of the extemporaneous pediatric oral formulations. Out of the 28 articles included in their study, 16 of them referred to one or many drug substances from the cardiovascular therapeutic area [21]. According to the literature, solid oral dosage forms are gaining popularity among children in terms of acceptability [36,37,38]. Regardless of the evident current shift towards the solid dosage forms, reflected also in the number of PIPs submitted [36], liquid oral dosage form still has the advantage of dose adaptation for different age categories and are still required for drug substances that are not approved in a solid oral dosage form. 

Considering this knowledge gap and the evident need for improvement in this area, the purpose of this study is to provide a comprehensive overview of the stability of liquid oral dosage form used in pediatric cardiology, with a particular focus on the requirements, the compounding practices, and the study design.

## 2. Materials and Methods

**Figure 1:**
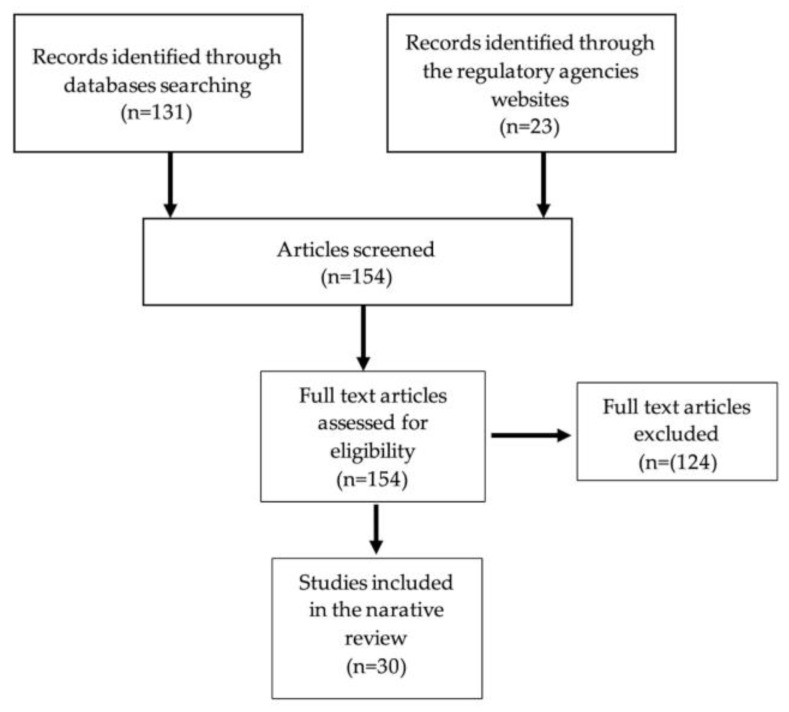
Flowchart describing literature search.

A comprehensive review of published papers on different databases (PubMed, ScienceDirect, PLoS One, and Google Scholar), using the search terms “pharmaceutical formulations in pediatric cardiology”, “pediatric oral solutions stability”, and “oral liquid dosage forms stability”, was performed from 5 September 2022 to 23 February 2023. The regulatory agencies’ websites of the FDA, EMA, and WHO, the International Consortium for Harmonization (ICH) guidelines, and the European Pharmacopoeia were also consulted. Inclusion criteria were studies in which the stability of oral dosage form was for cardiovascular substances. Following the search, the duplicates were removed. Abstracts were screened by two independent reviewers (C.-M.J.) and (G.J.) for eligibility of the predefined inclusion criteria. All disagreements were discussed with a senior author (S.I.). The selection process is described in Figure 1.

## 3. Results

### 3.1. Approved Medicines for Pediatric Use

In order to reflect the current status of the approved medication for pediatric therapeutic use, reports provided by the EMA and FDA have been consulted. There are several substances approved for cardiovascular therapy, either as new medicines, new indications, new pharmaceutical forms (EU), or based on which of them were granted pediatric labelling (US). A non-exhaustive list is provided in Table 1, based on reports provided by the EMA and FDA agencies [7,39]:

Based on the reports provided by the agencies, it can be observed that numerous active substances have undergone pediatric approval. However, in agreement with the findings of del Moral-Sanchez et al., few of the approved medicines are suitable for pediatric administration under the age of 6 years. This would imply the manipulation of the approved dosage form, which would also finally lead to off-label use.

### 3.2. General Considerations on Compounding and Stability of Liquid Oral Dosage Forms for Substances Used in Pediatric Cardiology

Pharmaceutical compounding plays a key role in providing individualized therapy for children [45] and it represents a safe alternative to the manipulation of adult-approved dosage forms. Pharmaceutical compounding is an accepted practice in some European countries and even in the USA [46,47]; however, the safety, efficacy, or quality of the finished product is not endorsed by a certain health authority. According to the FDA’s Human Drug Compounding Progress Report (2017), poor compounding practices associated with quality failure can cause serious patient injury or even death [48]. This concern has been raised recently in the literature, where attempts to standardize the processes and ensure their compliance with the regulatory requirements have been made [49,50,51].

**Figure 2:**
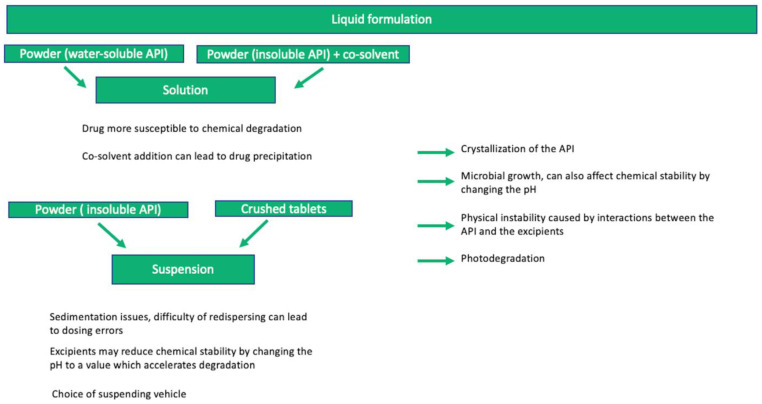
Left-hand side: stability issues for liquid dosage forms depending on the formulation (solutions or suspensions) and the compounding technique, respectively; right-hand side: common stability issues for liquid oral dosage forms.

All formulations used for extemporaneous preparation must be validated [52] and have supporting stability data. There are several well-known factors that influence stability, such as physicochemical interactions between the drug and the excipients, microbial growth, and chemical degradation of the API, which can negatively affect the quality and safety of a formulation. For exemplification purposes, factors that can generate stability issues in liquid oral dosage forms are illustrated in Figure 2.

It is fairly discussed in the literature that oral liquid dosage forms can have two provenances: API or crushed tablets, from commercially available adult formulations. While formulations obtained with the API can be more difficult to achieve due to the lack of availability of the API powder or solubility and compatibility issues, the formulations obtained via modification of a commercially available solid form (i.e., crushed tablets) can raise some concerns, such as the existence of potential harmful excipients for children, reduced solubility, difficulty to achieve a proper dosage both from stability and therapeutical perspectives, and interactions of the excipients with the API [53]. Another factor of concern is the trituration of an extended or controlled release tablet which can lead to dosing errors (immediate release of the entire API) or exacerbation of adverse effects [16]. Interestingly, a study conducted by Glass and Haywood reveals that out of 83 liquid formulations extemporaneously prepared by modifying the existing commercial dosage forms, only 7.2% presented stability concerns. The authors emphasize that taking into consideration the entire formulation along with potential interactions and degradation routes, instead of only considering the API characteristics, can lower the risks associated with these formulations, stability-wise [54].

With regards to the pediatric cardiovascular therapeutic area, Standing F and Tuleu C identified some problems related to the formulations: problems in dosing accuracy, unknown bioavailability of extemporaneous products, the use of potentially harmful excipients, and lack of access to modified release preparations [55]. Moreover, the cardiovascular drugs studied already present some particularities that make the preparation of a liquid oral dosage form quite challenging. Some examples are provided below:

- captopril generates captopril disulfide as a major degradation product; however, these processes are pH dependent [56], and some excipients can even enhance the degradation process [57];
- hydrochlorothiazide (rapid degradation to aminochlorobenzenedisulphonamide) [58];
- difficulty to achieve an adequate dosage and also to maintain stability at the same time [59];
- crystal formation [60];
- poor solubility [13,31,61];
- an unpleasant taste that would require the addition of sweeteners above the recommended range [31].

Due to these particularities, there are a limited number of formulations that can be chosen in order to ensure the stability of the final dosage form. For safety reasons, the use of a particular drug substance in compounding practices must undergo extensive research in order to determine if it is suitable for use. Many of the drug products marketed can change their approval status while the API and excipients necessary for the formulation can be readily available. In this sense, the FDA provides a list of drug products that cannot be compounded, mainly because the drug products in question have been removed from the market for safety and effectiveness reasons [62].

In general, it would be desirable to achieve a stable liquid formulation using a limited number of ingredients.

Another important point is represented by the excipients that are considered potentially harmful in children and especially in newborns. Ethanol, parabens, sodium benzoate, propylene glycol, and others are mentioned in the literature [18,63,64]. Regardless of the potential benefits for physicochemical and microbiological stability of liquid oral dosage forms, the EMA recommends the avoidance of the use particularly in pediatric formulations [18,65,66].

### 3.3. Stability-Indicating Parameters and Guidelines

According to the ICH Q1A (R2) quality guidelines, stability testing should be conducted to cover Critical Quality Attributes (CQA’s) that are susceptible to change during storage and are likely to influence the quality, safety, and/or efficacy: physical, chemical, biological and microbiological attributes, preservative content [67]. Regarding the test procedures and acceptance criteria for oral liquids or powders intended for reconstitution, ICH Q6A mentions the following applicable tests (one or more): uniformity of dosage units or weight variation, one or the other but not both; pH; microbial limits; antimicrobial preservative content; antioxidant preservative content (if applicable); extractables; alcohol content; dissolution; particle size distribution; redispersibility; rheological properties; reconstitution time; and water content for oral products that require reconstitution [68].

In the general monograph of liquid preparations for oral use, as well as for powders and granules for oral solutions and suspensions, the European Pharmacopoeia mentions the following tests to be conducted: uniformity of dosage units, uniformity of content, uniformity of mass and dose, and uniformity of dose of oral drops, respectively [69,70].

The fifty-second report of the World Health Organization (WHO) Expert Committee on Specification for Pharmaceutical Preparations, which is another guidance document referring to the stability of finished pharmaceutical products, indicates that regardless of the dosage form, appearance, assay, and degradation, products should be evaluated, along with preservative and antioxidant content, if applicable. In addition, the microbial tests should be performed at least at the beginning and the end of the stability test period. A non-exhaustive list of testing parameters for oral solutions, suspension, and emulsions is provided below:

- formation of precipitate, clarity (for solutions), pH, viscosity, extractables, and level of microbial contamination;
- additionally for suspensions, dispersibility, rheological properties, mean size, and distribution of particles should be considered. In addition, polymorphic conversion may be examined, if applicable [71].

The EMA also issued a guideline on the development of medicines for pediatric use. In this guideline, separate sections with instructions for liquid drug formulations are provided, such as oral suspensions and oral drops, and effervescent, soluble, and dispersible preparations, including considerations regarding packaging and measuring devices in order to avoid dosing errors [72].

As stated by the United States Pharmacopeia (USP) Compounding Expert Committee, a clear distinction should be made between strength testing, i.e., determining the amount of an active substance in a sample, and stability testing which is used to determine the shelf-life of a product. The stability must always be established by stability-indicating methods, and these comprise the method development, which usually consists of a forced degradation step, a method validation, and a properly designed stability study [73].

For the purpose of this review, the summary of the attributes chosen in the stability studies of liquid oral dosage forms conducted in the literature for the substances of interest is emphasized in Table 2.

Considering the CQAs to be verified for stability purposes highlighted by the regulators, the studies present in the literature indicate a general compliance with the ICH Q6A requirements. Physical, chemical, and microbiological tests were applied in order to verify the stability of liquid oral dosage forms, and the rheological properties, where applicable, for suspensions.

The organoleptic properties, such as appearance, odour, and colour, are strong indicators of the stability of the drug product and these are susceptible to change due to several factors: photosensitivity, microbial growth, and chemical interactions between the components [9]. Appearance testing is important also in suspensions, as it can indicate sediment formation [60]. Another reliable indicator of the stability of the oral liquid dosage forms is the pH level; changes in this parameter can severely affect the drug product solubility in the initial phase and the solution/suspension stability in the later phase. A change in pH can accelerate the degradation processes which could lead to the formation of certain potentially toxic degradation products above the accepted limit [58].

Regarding the chemical attributes, both ICH Q6A and European Pharmacopoeia mention the uniformity of dosage units as a test to be performed in the case of liquid oral dosage forms. Although this test is not mandatorily required in stability studies, it should be conducted as an initial test, taking into account the need for standardization of administered doses in liquid oral dosage forms. This test has been considered by a few studies [13,58,80].

In contrast, the assay of API and preservatives has been conducted for most of the studies. According to the USP, a stable extemporaneous product should preserve 90–110% of the initial content over the tested period [18,75]. These limits were set in most of the studies conducted; however, there were studies in which more restrictive limits were applied to preserve 95–105% of the initial content [86]. Tighter limits can be considered whenever a drug product demonstrates a robust stability profile and no decreasing tendency in the content of the liquid dosage form can be detected during the shelf-life.

Forced degradation studies were conducted in order to establish the stability-indicating method for most of the studies, which were developed or adapted from existing methods from the literature [60,80,92,93]. From forced degradation studies, several potential degradation products can be obtained which can or cannot be indicative of the actual degradation pathways of the drug product [19,94]. A stability-indicating method should demonstrate the full separation between the active substance and the main degradation products, using different chromatographic techniques [95,96,97] Nonetheless, the focus should be maintained on the major degradation products, and if these are not identified during stress testing, their quantification during stability studies is not deemed necessary [19]. Forced degradation has been conducted for most of the studies, and some of these followed the increase of degradation products over the tested period [75]. The primary degradation products should be defined and quantified during stability as a minimum standard of good practice, according to the FDA’s inspection guide for oral solutions and suspensions [98]. This guide is referring to drugs in the phenothiazines class, which shows evidence of instability. However, because chemical instability or degradation products appear [99] or increase over the testing period, which has been demonstrated for many other drug substances in the cardiovascular therapeutic area (e.g., captopril and amlodipine) [31,56], this recommendation should be extended to all solutions and suspensions. A plausible reason for which the quantification is often skipped is economic order since the acquisition of standards could be very expensive [77].

Concerning the liquid oral dosage forms formulated as suspensions or syrups, the redispersibility test should be applied as a minimum, if rheological properties and/or particle size distribution cannot be tested, although it has been emphasized in the literature that there is no official methodology describing this test in the guidelines [100]. The property of the active substance to resuspend after shaking will ensure content uniformity and avoid overdosing [101]. Rheological properties should be studied whenever possible, as increases in viscosity and particle size are directly related to sedimentation processes, and the rheological behavior can be influenced by many factors, such as the method of preparation, microbial growth, the ratio of excipients to drug substance, and interactions between vehicle and excipients [9,86,87].

Regardless of the dosage form (solution or suspension), microbial tests need to be performed in all stability studies, at least at the beginning and the end of the proposed shelf life, even though the formulation contains preservatives.

### 3.4. Stability Study Design

In the context of having limited stability data on a final product of liquid oral dosage form, a standard shelf life of 28 days or less is assigned and the expected duration of therapy should be considered [35]. According to the United States Pharmacopoeia, the beyond-use date assigned for liquid oral formulations is not later than 14 days when stored at controlled cold temperatures [102]. However, this approach is empirical considering that, as indicated in the paragraphs above, some drug substances may be more sensitive than others, or the choice of formulation (i.e., excipients and suspending base in case of suspensions) can significantly influence the stability of the formulation. Therefore, rigorous stability studies must be conducted in order to support an assigned shelf-life, for each particular formulation, with a combination of tablet/capsule and suspending medium, powder and different suspending medium, and different excipients used, considering the interactions that may occur [9].

For the purpose of this paper, the stability study design was considered taking into account the different factors that could influence the shelf-life of an oral liquid formulation, such as formulation and storage conditions. The results are summarized in Table 3.

In general, the studies conducted in the literature are well-designed, stability tests were conducted both at ambient room storage and in refrigerated conditions, given that different formulations will require different storage conditions. 

According to ICH Q1A (R2), long-term studies are carried out at 25 ± 2 °C/60% RH ± 5% RH or 30 ± 2 °C/65% RH ± 5% RH for a minimum of 12 months, and accelerated studies are carried out at 40 ± 2 °C/75 ± 5% RH for a minimum of 6 months. Drug substances that are intended for storage in a refrigerator are kept at 5 ± 3 °C for long-term storage for at least 12 months and at 25 ± 2 °C/60% RH ± 5% RH for 6 months, respectively, which represents the accelerated condition in this case. The testing frequency should be sufficient to establish a stability profile, e.g., at least 4 testing points in case of a product with a re-test period of 12 months (every three months in the first year, every 6 months in the second year, and then yearly through the proposed re-test period) [67]. 

Note, for the purpose of the paper, “long-term storage” was considered for any stability study conducted for more than 30 days, considering the usual short assigned shelf-life of the liquid oral dosage forms and the intended use of these (most are intended for hospital use). Nevertheless, valuable supportive information can be extracted for the solutions/suspensions stored for 12 months at long-term storage conditions and 6 months at accelerated conditions, respectively, with the proposed testing frequency as per guidelines or even more frequently. These studies demonstrate that with a wide selection of the formulation and excipients, the shelf-life of the oral liquid dosage form could be extended. Nevertheless, as stated throughout this paper, the use of such excipients that are potentially harmful to children should be avoided, as per guidelines [65]. Temperatures of storage were carefully chosen, yet relative humidity is mentioned in a few studies. One possible explanation could be that relative humidity is more relevant in solid oral dosage forms, where it can influence water uptake and enhance the degradation rate [103,104,105].

As part of stress testing, besides the forced degradation studies discussed in the paragraphs above, ICH Q1A (R2) [67] mentions photostability studies, which were conducted in even fewer studies compared to forced degradation. Supportive data can be found in the literature for some APIs describing the degradation pathways, photostability, and main degradation products, and this data should be used as a basis when considering the stability of the formulation [93,106,107,108,109,110]. Nevertheless, stress testing such as photostability has to be considered in APIs where there is little or no data available. 

As indicated in Table 3, most of the studies subjected multiple formulations to different storage conditions in order to either determine the influence of factors, such as vehicle type and excipients, on the stability of the final dosage form or to establish the best formulation in terms of stability. These studies assigned the shelf-life based on the most “promising” formulation, considering the use of certain excipients that could enhance their stability.

The aimed shelf-life was in most cases more than 30 days, which is acceptable in terms of storage at controlled temperature and humidity conditions, but in the case of liquid oral dosage forms, in-use studies are in fact the ones that best predict the stability behavior. Once the containers are opened, the stability of the formulation can be compromised by several factors; therefore, the physical, chemical, and especially microbiological stability must be tested under simulated conditions of in-use [89]. It is important to note that most of the extemporaneous liquid dosage forms used in the cardiovascular therapeutic area are intended to be used as multiple-dose; therefore, in-use studies would be prerogative. In-use studies were performed for a few oral liquid formulations [19,29,79]. The importance of performing in-use studies has also been emphasized by Standing J and Tuleu C, 2005 [55,111].

Regarding the storage conditions, room temperature (RT) storage seems to be the condition of choice. Refrigeration is often preferable with the scope of maximizing the chemical stability; however, precipitation of the active substance or the increase of viscosity can occur, limiting the shelf-life or even causing instability [60,112].

A discrepancy found in the compounding technique between the studies refers to the use of the suspending vehicles. While the new commercially available suspending bases can ease a lot the compounding process and provide liquid dosage forms of acceptable stability, being physically and chemically compatible with a wide range of APIs [113,114], most of the studies used classical vehicles in the formulation of the final liquid dosage forms. An advantage of modern suspending bases would be the compounding technique and the stability provided by the manufacturer. This topic has also been considered by Thrimawithana et al., comparing the classical and modern suspending bases, and concluded that there are APIs for which traditional compounding was more suitable [115].

When a longer shelf-life is aimed, the compatibility of the container with the liquid formulation should also be assessed on a case-by-case basis, and this can be demonstrated during stability tests under normal conditions of use [116]. It is a known fact in stability that the packaging material could possibly influence the stability of a finished dosage form. The European Pharmacopoeia highlights some aspects to be considered regarding the container of choice, but one of the most important is that the container, either glass or plastic, must not interfere with the stability of the formulation or release substances that can be potentially toxic [69,70]. However, this aspect has not been considered in the literature so far for liquid formulations, even though the studies aimed for a longer shelf-life (e.g., 6 months, 12 months). A possible explanation could be that the studied formulations presented acceptable stability in either plastic prescription bottles or glass containers, and the choice of packaging was made depending on the designated use (i.e., ambulatory setting or hospital setting). One study even proposed the influence of the packaging material (i.e., PET or glass containers), yet did not provide further information since the studied formulation presented instability due to particle formation in both studied packaging [34]. The packaging as a possible influence factor for stability was also emphasized by Haywood and Glass 2013, reporting different shelf-life in plastic syringes versus glass bottles of the same formulation [116].

Many approaches have been taken recently in order to achieve stable formulations and to optimize the manner in which solubilization and bioavailability are achieved. For instance, Cirri et al. studied the influence of cyclodextrin complexation and nanolipid carriers for obtaining a stable and effective liquid formulation of hydrochlorothiazide. Phase-solubility studies, dissolution rate studies, drug release studies, stability under gastric conditions, and in vivo studies have been conducted [82]. This demonstrates a clear tendency of the research towards obtaining stable and safe formulations for pediatric use. Moreover, the bioavailability studies are a step forward in gathering information on how effective the drug products used in pediatrics are exactly.

Another interesting approach in the stability study design of oral liquid dosage forms is to apply the principles of Quality by Design (QbD) in order to determine the most suitable formulation from a stability perspective [117]. Such cases have demonstrated, by extrapolation, chemical stability for at least 850 days. The limitations would be represented by the microbial enumeration tests, which failed to meet the specifications after 180 days [12].

## 4. Conclusions

Overall, this study highlights several aspects that should be considered when conducting a stability study for liquid oral dosage forms, referring not only to the guidelines and the regulations but also to the particularities of drug substances used in cardiovascular pharmacotherapy.

While the stability studies are found to be conducted in an acceptable manner, both for parameters tested and stability study design, there is an evident opportunity for improvement. Altogether, the information gathering and the results of the studies can be translated into clinical practice in order to achieve the desired quality of liquid oral dosage forms.

Ultimately, considering the number of studies present in the literature regarding this topic and the regulations in place, this paper emphasizes the continuous need for oral liquid dosage forms in pediatrics and especially in pediatric cardiology, despite the current evident shift towards solid oral dosage forms.

## Figures and Tables

**Table 1:** Approved medicines for pediatric use.

Active Substance	Age	Type of Approval	Dosage Form	Indication	Year of Approval
Bosentan [40]	1–15 y	New medicine including pediatric indication	Film-coated tablets	PAH	2013
Propranolol [41]	5 w–5 m		Oral solution	Hemangioma	2014
Nitric oxide [42]	0–17 y	Extension (new pediatric indication)	Gas for inhalation	PAH	2011
Sildenafil [43]	1–17 y		Film-coated tablets Solution for injection Powder for oral suspension	PAH	2011
Bosentan [44]	3 m–18 y	Extension of indication to include new age group	Film-coated tablets	PAH	2015
Nitric oxide	0–17 y	Addition of a new dosage strength	Gas for inhalation	PAH	2011
Amlodipine ^1,2^	6–17 y	Nationally authorized medicine (HU)	Film-coated tablet	hypertension	2011
Losartan ^1^	2.5 mg/mL 6–16 y	Nationally authorized medicine (RO, HU)	Powder for oral suspension	hypertension	2009/2011
Losartan ^1^		New pediatric indication (RO, IT, FI)	NS		2009/2010
Losartan ^1^		New pharmaceutical formulation (CY, EE, IT, ES, UK)	Powder for oral solution/suspension		2009
Spironolactone ^1,2^	25 mg, 50 mg	Nationally authorized medicine (HU)	Film-coated tablet	Congestive heart failure	2011
Valsartan ^2^	3 mg/mL 1–18 y	Nationally authorized medicine (HU, CZ)	Oral solution	Hypertension	2010
Valsartan ^2^		New formulation (AU, CY, CZ, EE, FI, IT, RO, SI, ES, SW, UK)	Oral solution/divisibility of the tablet		
Valsartan ^2^	6–18 y	New pediatric indication (CY, EE, RO, FI, SW)	Film-coated tablet	Hypertension	2010
Candesartan ^2^	6–18 y	New pediatric indication (CY)	Film-coated tablet	Hypertension	2013
Levamlodipine	6–17 y	Labelling change	Tablet	Hypertension	2019
Amlodipine benzoate	>6 y	Labelling change	Oral suspension	Hypertension	2019
Aliskiren	>6 y	Labelling change	Pellets	Hypertension	2017
Sodium nitroprusside	NS	Labelling change	Injectable	Hypertensive crisis	2013
Olmesartan	>6 y	Labelling change	Tablet	Hypertension	2010
Candesartan	1–17 y	Labelling change	Tablet		2009
Eplerenone	4–17 y ^3^	Labelling change	Tablet		2008
Valsartan	6–16 y	Labelling change	Tablet		2007
Metoprolol	6–16 y ^3^	Labelling change	ER Tablets		2007
Carvedilol	2 m–17 y	Labelling change	Tablet	Heart failure	2007
Irbesartan	6–16 y ^3^	Labelling change	Tablet	Hypertension	2006
Fenoldopam	<1–12 y	Labelling change	Injectable	In hospital, short-term reduction of hypertension	2004
Losartan	6–16 y	Labelling change	Tablet	Hypertension	2004
Amlodipine	6–17 y	Labelling change	Tablet		2004
Lisinopril	6–16 y	Labelling change	Tablet		2003
Fosinopril	6–16 y ^3^	Labelling change	Tablet		2003
Sotalol	3 d–12 y ^3^	Labelling change	Tablet	Arrhythmia	2001
Enalapril	1 m–16 y	Labelling change	Tablet	Hypertension	2001

^1^—Summary of product characteristics not found on EMA or national database; ^2^—summary of product characteristics available on the National Medicines Agency from Romania database; ^3^—based on the data gathered from clinical trials; y—year(s); m—month(s); w—week(s); d—day(s); PAH—Pulmonary Arterial Hypertension; HU—Hungary; RO—Romania; IT—Italy; FI—Finland; CY—Cyprus; EE—Estonia; ES—Spain; UK—United Kingdom; CZ—Czech Republic; AU—Australia; SI—Slovenia; SW—Sweden; NS—not specified.

**Table 2:** Stability-indicating parameters.

Stability Testing									
	Appearance/Organoleptic Properties	pH	Assay of API/Preservatives	Degradation Products	MT	Redispersibility	Rheological Properties	Particle Size Distribution	Ref.
Amlodipine besylate 1 mg/mL	+	+	+	+	−	−	−	−	[74]
Amlodipine besylate ^&^ 0.5 mg/mL and 10 mg/mL	+	+	+	+	−	−	+	−	[75]
Amlodipine besylate 0.5 mg/mL	+	+	+	+	+	NA	NA	NA	[31]
Amlodipine/Valsartan 5/80 mg/5 mL ^	+	+	+	−	+	−	+	−	[29]
Atenolol	+	+	+	−	+	−	−	−	[12]
Candesartan 1 mg/mL	+	−	+ **	−	− ^#^	+	+	+	[9]
Valsartan 4 mg/mL									
Captopril 1 mg/mL and 5 mg/mL	+	+	+	+	+	NA	NA	NA	[76]
Captopril 1 mg/mL	+	−	+	+	+	NA	NA	NA	[77]
Carvedilol + CD 5 mg/mL	−	−	+	+	−	NA	NA	NA	[61]
Carvedilol 1 mg/mL	−	−	+	+	−	−	−	−	[78]
Clonidine 50 μg/mL	+	+	+	−	−	NA	NA	NA	[59]
Clonidine 10 μg/mL	+	+	+	+	+	NA	NA	NA	[19]
Clonidine 20 μg/mL	+	+	+	+	+	NA	NA	NA	[79]
Flecainide acetate 10 mg/mL	+	+	+ *	−	+	NA	NA	NA	[60]
Flecainide acetate 20 mg/mL									
Flecainide 20 mg/mL	+	−	+ **	−	+	+	+	−	[80]
Chlorothiazide 10 mg/mL + Furosemide 1 mg/mL	+	−	+	+	−	NA	NA	NA	[81]
Furosemide 5 mg/mL	−	+	+	−	+	+	−	+	[58]
Spironolactone 5 mg/mL									
Hydrochlorothiazide 5 mg/mL									
Furosemide 2 mg/mL	+	+	+	+	+	NA	NA	NA	[15]
Spironolactone 5 mg/mL	+	+	+ **	+	−	NA	NA	NA	[18]
Hydrochlorothiazide 2 mg/mL									
Hydrochlorothiazide + CD 2 mg/mL ^	−	−	−	−	−	−	+	+	[82]
Lisinopril 1 mg/mL	+	+	+	−	−	−	−	−	[83]
Losartan 5 mg/mL	+	+	+	+	+	+	−	−	[84]
Nicardipine 2 mg/mL	+	+	+ **	−	+	NA	NA	NA	[13]
Nifedipine 4 mg/mL	+	+	+	+	−	+	−	−	[85]
Propranolol 2 mg/mL and Propranolol 5 mg/mL	+	+	+	−	+	+	+	−	[86]
Ramipril 1 mg/mL	+	+	+	+	−	+	+	+	[87]
Sildenafil 2 mg/mL	+	+	+	+	+	+	+	+	[88]
Sildenafil 2.5 mg/mL	+	+	+	+	+	+	+	−	[89]
Sotalol 5 mg/mL	+	+	+	−	−	NA	NA	NA	[90]
Sotalol 5 mg/mL	+	+	+	−	−	+	−	−	[91]

+—tested; −—not tested; *—takes into consideration the need for preservative to be added to the formulation; ^—drug release study was also carried out for this suspension; &—bracketing approach used; #—changes in the solution smell and taste were indicators of a possible microbial growth; **—mass uniformity and/or uniformity of dosage units also evaluated; NA—not applicable.

**Table 3:** Stability study design.

Design of Stability Study								
Drug Substance Dosage Strength	Formulation/ Number of Formulation	Source for Formulation/Vehicles	Study Type *	Duration	Storage Conditions	Testing Frequency	Assigned Shelf-Life	Ref.
Amlodipine besylate 0.5 mg/mL	Solution/3	Crushed tablets/Purified water	Long-term	12 months	25 ± 2 °C	0, 1, 2, 3, 6 months	NS	[31]
					40 ± 2 °C			
			Accelerated					
			In-use		4 ± 2 °C	0, 1, 2, 3, 6 9, 12 months		
Amlodipine besylate 1 mg/mL	Suspension/2	Crushed tablets/MC + syrup,	Long-term	91 days	4 °C	T0, 7, 14, 28, 42, 56, 70, 91 days	91 days refrigeration	[74]
		OraPlus^®^ + OraSweet^®^	Accelerated	56 days	25 °C			
Amlodipine besylate 0.5 mg/mL and 10 mg/mL ^#^	Suspension/2	Powder/SuspendIt base	Long-term	180 days	5 °C	T0, 7, 14, 29, 46, 60, 90, 120, 180 days	90 days	[75]
					25 °C		7 days	
Amlodipine/Valsartan 5/80 mg/5 mL	Suspension/1	Crushed tablets/guar gum	Long-term	4 weeks	RT	T0, week 1, week 2, week 3, week 4	4 weeks RT	[29]
Atenolol 1% and 4%	Syrup/3	Powder/Syrup, Glycerin, or both	Long-term	6 months	4 ± 2 °C	T0, 6 months	6 months	[12]
					25 ± 2 °C/60 ± 5%RH		3 months ^@^	
			Accelerated					
					40 ± 2 °C/75 ± 5%RH		6 months	
Candesartan 1 mg/mL	Syrup/3	Crushed tablets/Xanthan gum, vehicle for oral solution USP, sucrose syrup	Long-term	35 days	25 °C	T0, 7, 14, 28, 35 days	14 days ^@^	[9]
Valsartan 4 mg/mL	Syrup/3				4 °C			
Captopril 1 mg/mL and 5 mg/mL	Solution/1	Powder/Sterile water for irrigation	Long-term	12 months	22 °C	T0, 3, 6, 9, 12 months	12 months	[76]
			In-use	1 month	4–8 °C	T0, 1 month		
Captopril 1 mg/mL	Solution/4	Powder/Preservative solution	Long-term	39 days	22 °C	NS	7 days	[77]
					t < 8 °C			
Carvedilol + CD 5 mg/mL	Solution/2	Powder/γCD or RAMEB in aqueous media	Long-term photostability	6 months	25 °C/60%RH + 7500 Lux and UV light	T0, 1, 2, 3, 6 months	6 months	[61]
Carvedilol 1 mg/mL	Solution/2	Powder/Propylene glycol and Polyvinylpyrrolidone	Long-term	56 days	4 °C	T0, 3, 7, 14, 28, 56 days	56 days at RT	[78]
					25 °C			
			Accelerated					
					40 °C			
Carvedilol 1 mg/mL	Suspension/1	Powder/aqueous suspension vehicle	Long-term	56 days	4 °C			
					25 °C			
			Accelerated		40 °C			
Clonidine 50 μg/mL	Solution/1	Powder/distilled water	Long-term	9 months	RT	T0, 1, 2, 3 6, 9 months	9 months	[59]
Clonidine 10 μg/mL	Solution/1	Powder/Inorpha^®^	Long-term	60 days	5 ± 3 °C	T0, 15, 21, 30, 60 days	60 days	[19]
			In-use		25 ± 2 °C		~	
					In-use	36, 45, 51, 60 days	5 ± 3 °C p.f.l	
Clonidine 20 μg/mL	Solution/2	Powder/Purified water + simple syrup	Long-term	90 days	5 ± 3 °C	T0, 2, 6, 10, 14, 20, 30, 40, 50, 70, 90 days	90 d	[79]
					25 ± 2 °C			
					40 ± 2 °C			
			In-use					
					In-use	T0, 7, 14, 28, 42 days	5 ± 3 °C p.f.l	
Flecainide acetate 10 mg/mL	Solution/8	Powder/Ultra pure water	Long-term	56 days	4 ± 1 °C	T0, 14, 28, 42, 56 days	&	[60]
Flecainide acetate 20 mg/mL					25 ± 1 °C		8 weeks	
					40 ± 1 °C		/	
Flecainide 20 mg/mL	Solution/4	Powder/Simple syrup	Long-term	60 days	5 ± 0.1 °C	T0, 15, 30, 15 for physicochemical	NS	[80]
					25 ± 0.1 °C	T0, 10, 30, 60 for microbiology		
					40 ± 0.1 °C			
Chlorothiazide 10 mg/mL + /Furosemide 1 mg/mL	Solution/2	Powder/ Sol. for inj/Dextrose 5% USP	Photostability	96 h	25 °C/60% RH	T0, 24, 36, 48, 72, 96 h	96 h p.f.l	[81]
Furosemide 5 mg/mL	Suspension/1 Suspension/2 for Hydrochlorothiazide	Powder/Aqueous suspension vehicle based on CMC Na as suspending agent	Short-term	7 days	25 ± 2 °C	T0, 7 days	7 days; NS	[58]
Spironolactone 5 mg/mL								
					5 ± 3 °C			
Hydrochlorothiazide 5 mg/mL								
Furosemide 2 mg/mL	Solution/2	Powder/Water for injection	Long-term	9 months	25 ± 3 °C	T0, 7, 30, 90, 180, 270 days	9 months RT	[15]
					40 ± 0.5 °C			
Spironolactone 5 mg/mL	Suspension/1	Powder/Syrspend^®^PH4 Dry	Long-term	60 days	22 ± 4 °C	T0, 7, 14, 30, 42, 60 days	60 days ^@^	[18]
Hydrochlorothiazide 2 mg/mL					5 ± 3 °C			
Hydrochlorothiazide + CD 2 mg/mL	Solution/2	Powder/HPβCD or SBEβCD and Nano-lipid carriers	Long-term	3 months	4 °C	T0, 1, 2, 3 months	NS	[82]
Lisinopril 1 mg/mL	Suspension/2	Crushed tablets/MC 1% Syrup/OraPlus^®^ + OraSweet^®^	Long-term	91 days	25 °C	T0, 7, 14, 28, 42, 56, 70, 91 days	NS	[83]
					4 °C in plastic prescription bottles			
Losartan 5 mg/mL	Suspension/2	Crushed tablets/OraPlus^®^ +OraSweet^®^/Deionised water	Long-term	28 days	4 °C	T0, 1, 3, 7, 10, 14, 21, 28 days	28 days	[84]
					RT			
Nicardipine 2 mg/mL	Solution/3	Powder/InOrpha^®^ ***/Orablend^®^/Syrspend SF^®^	Long-term	90 days	2–8 °C	T0, 1, 2, 7, 15, 30, 60, 90 days	90 days	[13]
					25 °C			
Nifedipine 4 mg/mL	Suspension/2	Capsules/MC 1% Syrup/OraPlus^®^ + OraSweet^®^	Long-term	91 days	4 °C	T0, 7, 14, 28, 42, 56, 70, 91 days	NS	[85]
					25 °C/60 °C			
Propranolol 2 mg/mL and 5 mg/mL	Suspension/1	Crushed tablets or Powder/OraSweet^®^/Vehicle for oral solution USP/Sucrose syrup	Long-term	35 days	4 °C	T0, 35 days	35 days	[86]
	Syrup/2				25 °C			
Ramipril 1 mg/mL **	Solution/2	Powder/Acetic acid/HPβCD/Xanthan Gum	Accelerated	6 months	40 °C/75%RH	T0, 12 months ^	^@^	[87]
	Suspension/1		Long-term	12 months	25 °C/60%RH		12 months	
Sildenafil 2 mg/mL	Suspension/1 Solution/2	Powder/Bidistilled water	Long-term	90 days	4 °C	T0, 7, 15, 30, 60, 90 days	15 days	[88]
					25 °C		90 days	
					40 °C		60 days	
Sildenafil 2.5 mg/mL	Suspension/1	Crushed tablet/1% MC and syrup	Long-term	90 days	30 °C/75%RH	T0, 7, 14, 21, 28, 60, 90 days	90 days	[89]
			Accelerated		40 °C/75%RH			
			In-use		RT	T0, 7, 14	14 days	
Sotalol 5 mg/mL	Solution/3 ****	Powder/Water for injection	Long-term	180 days	25 ± 2 °C	T0, 7, 14, 30, 60, 90, 120, 150, 180 days	180 days under refrigeration	[90]
					5 ± 3 °C			
Sotalol 5 mg/mL	Suspension/2	Crushed tablets/MC 1% Syrup/OraPlus^®^ + OraSweet^®^	Long-term	91 days	4 °C	T0, 7, 14, 28, 42, 56, 70, 91 days	3 months ^^	[91]
					25 °C			
					25 °C			

*—long-term is considered at least 30 days, which would ensure a monthly treatment, in case of chronic treatment; **—defined in the study as “not more than 5 mL to deliver 5 mg”; ^—tested on a monthly basis, from the beginning until the end of the study; NS—assigned shelf-life or storage conditions not specified; CD—cyclodextrin; #—bracketing approach used; @—Assigned shelf-life limited by microbial growth/failure to meet the acceptance criteria for drug content/changes in organoleptic properties; ~—a 30-day shelf-life was not supported due to decrease in content; &—refrigeration should be avoided due to crystal formation; ***—this vehicle was the only one demonstrating stability; therefore, studies were continued with only one formulation; T0 represents the initial testing; ****—one of the formulations did not achieve stability; ^^—longer shelf-life not supported by possible microbial growth; NA—stability not achieved with neither one of the formulations; RT—room temperature; RH—relative humidity.

## Data Availability

Not applicable.
